# Relationship between coccolith length and thickness in the coccolithophore species *Emiliania huxleyi* and *Gephyrocapsa oceanica*

**DOI:** 10.1371/journal.pone.0220725

**Published:** 2019-08-05

**Authors:** Simen Alexander Linge Johnsen, Jörg Bollmann, Christina Gebuehr, Jens O. Herrle

**Affiliations:** 1 Department of Earth Sciences, University of Toronto, Toronto, Ontario, Canada; 2 Institute of Geosciences, Goethe-University Frankfurt, Frankfurt am Main, Germany; 3 Biodiversity and Climate Research Centre (BIK-F), Frankfurt am Main, Germany; Helmholtz Centre for Ocean Research Keil, GERMANY

## Abstract

Coccolith mass is an important parameter for estimating coccolithophore contribution to carbonate sedimentation, organic carbon ballasting and coccolithophore calcification. Single coccolith mass is often estimated based on the k_s_ model, which assumes that length and thickness increase proportionally. To evaluate this assumption, this study compared coccolith length, thickness, and mass of seven *Emiliania huxleyi* strains and one *Gephyrocapsa oceanica* strain grown in 25, 34, and 44 salinity artificial seawater. While coccolith length increased with salinity in four *E. huxleyi* strains, thickness did not increase significantly with salinity in three of these strains. Only *G. oceanica* showed a consistent increase in length with salinity that was accompanied by an increase in thickness. Coccolith length and thickness was also not correlated in 14 of 24 individual experiments, and in the experiments in which there was a positive relationship r^2^ was low (<0.4). Because thickness did not increase with length in *E. huxleyi*, the increase in mass was less than expected from the k_s_ model, and thus, mass can not be accurately estimated from coccolith length alone.

## Introduction

Anthropogenic emission of CO_2_ into the atmosphere is increasing CO_2_ levels in both the atmosphere and ocean at an unprecedented rate [1]. This rapid increase of CO_2_ is affecting the carbon chemistry of the surface ocean, leading to a pH decrease through a process called ocean acidification [2]. Ocean acidification may affect calcification in several marine organism groups (e.g. [3–5]), including coccolithophores (e.g. [6]).

Coccolithophores are an important marine group of single celled calcifying algae characterized by the production of calcitic plates called coccoliths. As both primary and calcite producers, coccolithophores play a dual role as a CO_2_ sink and source in the ocean surface [7–9]. Moreover, single coccolith mass is an important factor of the oceanic carbonate budget [8, 10], and for the formation of ballast in sinking aggregates which aid drawdown of organic carbon in the ocean [11–13]. The impact of ocean acidification on calcification in this group has therefore generated much interest over the past two decades. Several studies have reported conflicting data on the influence of seawater carbon chemistry on coccolithophore calcification in laboratory (e.g. [6, 14]), mesocosm (e.g. [15, 16]), and field studies (e.g. [17–19]). These conflicting results may be rooted in problems with the application of the methods used to quantify coccolithophore calcification.

Several methods exist to estimate coccolithophore calcification. The simplest method is just weighing how much calcite and organic carbon were produced over time in a culturing experiment [20]. However, such weighing may not be used for obtaining species-specific data in plankton or sediment samples, and field studies have therefore used different approaches for estimating coccolithophore calcification. For example, the qualitative visual inspection of the overall calcification of coccoliths from SEM images (e.g. [18, 21, 22]), the estimation of single coccolith mass as measured from coccolith interference colours (e.g. [23, 24]), or the estimation of mass from coccolith length (e.g. [25, 26]).

Recent studies revealed the limitations and sources of error for coccolith mass estimates from interference colours under a light microscope (see [24, 27–31] for details). However, the commonly used volumetric method for estimating mass by [26] has not yet been evaluated. This method uses coccolith length and a volumetric k_s_ model to relate coccolith length to mass using the equation below [26]:

$$\begin{array}{c} m=k_{s}\times l^{3}\times d \end{array}$$ (1)

where *m* is coccolith mass, *l* is coccolith length, *d* is density of calcite (= 2.71 pg μm^−3^), and *k_s_* is a species-specific shape constant. This model assumes isometric coccolith growth [26], i.e. that length and thickness grow at proportionate rates.

Several studies have used the k_s_ model to estimate coccolith contribution to carbonate sedimentation (e.g. [32, 33]), and it is therefore important to validate the model assumptions to assess the accuracy of this method. However, the assumption that coccolith length and thickness increase at a constant rate has yet to be tested, and might reflect a major source of error when estimating coccolith mass from length. The goal of this study is therefore to evaluate the assumption that the thickness of coccoliths increases with increasing length at a constant rate. To achieve this, strains of the two species *Emiliania huxleyi* and *Gephyrocapsa oceanica* were grown under different salinity conditions, as salinity is known to affect *E. huxleyi* coccolith length (e.g. [34–39]). By measuring coccolith thickness with increasing coccolith length, the relationship between coccolith length and thickness in *E. huxleyi* and *G. oceanica* could be investigated and the usefulness of the k_s_ model for estimating the mass of these two species evaluated.

## Materials and methods

**Fig 1:**
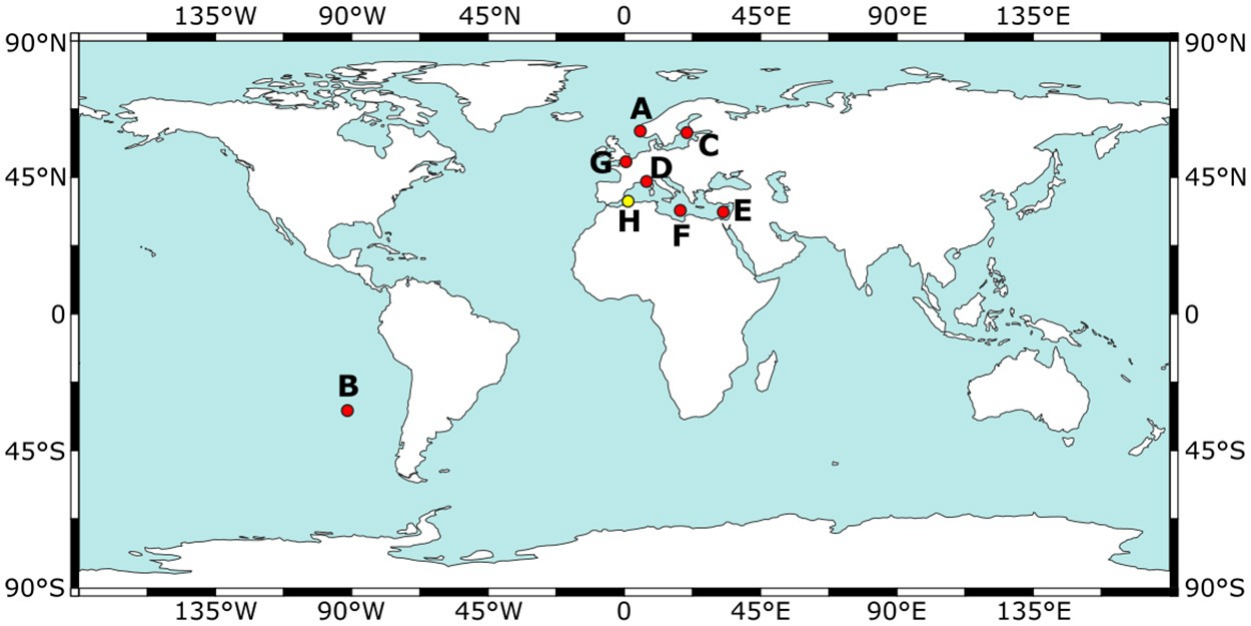
Location of isolation sites for each strain used in this study. Red circles: *E. huxleyi* strains (A: PLY B11. B: RCC 868. C: RCC 1210. D: RCC 1232. E: RCC 1824. F: RCC 1843. G: SAG 33.90); Yellow circle: *G. oceanica* strain (H: RCC 1223). World map was made with Natural Earth.

Seven *Emiliania huxleyi* strains (Morphotype Type A [40]) and one *Gephyrocapsa oceanica* (Morphotype *Gephyrocapsa* larger [41]) strain were used for this study (see Table 1 and Fig 1 for details). *E. huxleyi* strains were originally isolated from sites with salinities ranging from ~20 to ~39 and include both coastal and open ocean clones.

**Table 1:** Information on coccolithophore strains used in this study, including location, year of isolation, and in situ salinity.

Strain	Species	Morphotype	Isolation year	Ocean/Sea	Country	Latitude	Longitude	Salinity
PLY B11	*E. huxleyi*	A	1992	North Sea	Norway	60°18’N	05°15’E	~30
RCC 868	*E. huxleyi*	A	2004	South East Pacific	Chile	31°41’S	91°29’W	~34
RCC 1210	*E. huxleyi*	A	1998	Baltic Sea	Sweden	59°77’N	20°64’E	~20
RCC 1223	*G. oceanica*	Larger	1999	Mediterranean Sea	Spain	37°10’N	01°13’E	~37
RCC 1232	*E. huxleyi*	A	N/A	Mediterranean Sea	France	43°41’N	07°19’E	~37
RCC 1824	*E. huxleyi*	A	2008	Mediterranean Sea	Cyprus	33°37’N	32°39’E	~39
RCC 1843	*E. huxleyi*	A	2008	Mediterranean Sea	Libya	34°08’N	18°27’E	~38
SAG 33.90	*E. huxleyi*	A	1950	North Sea	United Kingdom	50°11’N	00°30’E	~34

### Culture preparation

Culture medium was prepared from artificial seawater with deionized water and three different concentrations of synthetic sea salt (Ultramarine, Waterlife Research Industries, Longford, UK) to get water with three different salinities of 24.7, 34.3 and 44.3 units. Salinity was measured using a WTW Multi 3400i handheld conductivity meter (Xylem Analytics Germany Sales GmbH & Co., Wellheim, Germany). 0.5 g L^−1^ Tricene was added to each solution to prevent precipitation of the salt during the subsequent autoclaving. Sterile f/2 nutrient solution [42] was added to each culture medium after autoclaving. Each algal strain was kept under a 12:12 light:dark cycle at 15°C and a salinity of ~35 before inocculation. 8 × 10^6^ cells of each strain were then inocculated in 20 mL flasks for each of the three salinity conditions by mixing 1.6 to 2.8 mL of the original algal medium with 17.2 to 18.4 mL of the prepared culture medium for initial cell concentrations of ~4.0 × 10^5^ cells/mL in each sample. Each sample was grown at 15°C under a white fluorescence lamp set to 50-200 μmol photons m^−2^ s^−1^ with a correlated colour temperature of 6500K using a continuous light cycle. Cell density and cell size was monitored daily using a CASY Model TT cell counter (Roche Diagnostics, Risch-Rotkreuz, Switzerland) by sampling 400 μL of each experiment and mixing with 10 mL freshly filtered CASY ton. The samples were all grown for nine to eleven days until the late exponential growth phase. Growth rate during the exponential phase was calculated as:

$$\begin{array}{c} \mu =\frac{ln\left(c_{1}\right)-ln\left(c_{0}\right)}{t} \end{array}$$ (2)

where *μ* is growth rate in day^−1^, *c_0_* is cell density at start of the exponential growth phase, *c_1_* is cell density at end of the exponential growth phase and *t* is the duration of the exponential growth phase in days. When the coccolithophore experiments reached the late exponential growth phase, 5 mL of each experiment were filtered on 0.4 μm pore size polycarbonate filters using a vacuum pump. At the time of sampling, cell densities ranged from 3.3 × 10^5^ cells/mL to 2.5 × 10^6^ cells/mL (Table 2).

**Table 2:** Measured growth rate (μ) and cell density at time of sampling for strains at 25, 34, and 44 salinity and 15°C.

Strain	Species	μ (day^-1^)			Cell density at end of experiment (cells/ml)		
		25 salinity	34 salinity	44 salinity	25 salinity	34 salinity	44 salinity
PLY B11	*E. huxleyi*	0.194	0.135	0.184	1.0 X 10^6^	9.4 X 10^5^	1.6 X 10^6^
RCC 868	*E. huxleyi*	0.182	0.402	0.223	1.8 X 10^6^	7.2 X 10^5^	1.5 X 10^6^
RCC 1210	*E. huxleyi*	0.286	0.601	0.207	1.1 X 10^6^	2.1 X 10^6^	1.7 X 10^6^
RCC 1223	*G. oceanica*	0.240	0.065	0.170	1.6 X 10^6^	1.4 X 10^6^	2.5 X 10^6^
RCC 1232	*E. huxleyi*	0.226	0.302	0.191	1.4 X 10^6^	9.3 X 10^5^	2.4 X 10^6^
RCC 1824	*E. huxleyi*	0.184	0.371	0.193	9.4 X 10^5^	3.3 X 10^5^	1.4 X 10^6^
RCC 1843	*E. huxleyi*	0.234	0.038	0.089	1.0 X 10^6^	5.3 X 10^5^	5.5 X 10^6^
SAG 33.90	*E. huxleyi*	0.409	0.313	0.245	1.7 X 10^6^	6.5 X 10^5^	2.2 X 10^6^

### Imaging

For visual assessment of samples in a Scanning Electron Microscope (SEM), a triangular piece was cut from each filter membrane, mounted on an aluminum stub, and coated with 3nm of platinum using a Leica SCD500 Metal Coater (Leica Microsystems, Wetzlar, Germany). SEM images with 1024 x 768 pixels were then captured from each sample at 16,000x or 20,000x magnification using a Zeiss Supra VP55 SEM (Carl Zeiss, Oberkochen, Germany) for visual evaluation of the coccoliths. The resolution was ~2nm and the geometry and accuracy of size measurements performed with the SEM in this study were controlled by measuring about 30 mono-sized polymer calibration spheres with a diameter of 2 μm (DYNO Particles AS, Norway, product no. SS-15-PXG).

Coccoliths from each sample were transferred from their filters onto a glass slide and mounted using NOA 61 adhesive (Norland Inc., Cranbury, New Jersey, USA) for light microscope (LM) analysis. Imaging was done with a Zeiss Axio Imager Z1 light microscope (Carl Zeiss, Oberkochen, Germany) equipped with a 1.6x optovar, neutral density filters, a Plan-Apo 100x, 1.4 NA oil objective, a 0.9 NA universal condenser, a Benford plate for circular polarization [43], and a Canon 60D DSLR camera (Canon Inc., Tokyo, Japan) for digital imaging. The light microscope and camera were calibrated for accurate retardation measurements according to the Circular Polarizer Retardation estimates (CPR) method [24], using a calibration curve obtained from a recent revision of the Michel-Lévy chart [44] and two polymer films of a known retardation (31nm and 129nm). The condenser was partly closed to avoid polarization aberrations [45, 46]. 30 coccoliths from each sample were captured in RAW format with a 5194 x 3457 pixel resolution at 160x magnification for a pixel size of 0.0003 μm^2^. A S8 Stage micrometer (02A00404, PYSER-SGI Ltd., Edenbridge, UK), in steps of 10 μm along a line with a total length of 1000 μm ±1 μm, was used for size calibration.

### Coccolith measurements

The length, thickness, and mass of 30 coccoliths per sample were measured using the CPR-method [24]. RAW images were converted to colour corrected TIFF in sRGB colour space with an applied gamma of 2.2 [44]. Subsequently, images were converted to 8-bit images and coccoliths were segmented from the background in ImageJ 1.52g using a Canny-Deriche edge detection algorithm [47, 48]. The function [Calibrate…] of ImageJ was used to relate grey values of a Michel-Lévy chart [44] to coccolith pixel mass. Coccolith measurements were then obtained using the ImageJ function [Analyze Particles…]. Each measurement is related to multiple sources of uncertainty. For example, variation in light intensity, different fits to calibration curves, and curve resolution are sources of error for thickness. Uncertainties were therefore calculated by error propagation to give a standard uncertainty of each measurement at a 95% confidence level [49]. The standard uncertainty of each measurement at 95% confidence level is ±0.2 μm for length, ±0.007 μm for thickness, and ±~13-20% for mass (depending on particle size; ~0.1-0.2pg for *E. huxleyi* and ~0.9pg for *G. oceanica*). Differences in length, thickness, and mass can thus only be fully resolved when they are at least 0.4 μm, 0.014 μm, and 0.2-0.4pg (for *E. huxleyi*) or 1.8pg (for *G. oceanica*), respectively.

The length of 30 coccoliths from each of the PLY B11 cultures grown at 25 and 34 salinity were also measured from SEM images to confirm measured lengths in the LM.

#### Coccolith skewness and aspect ratio

**Fig 2:**
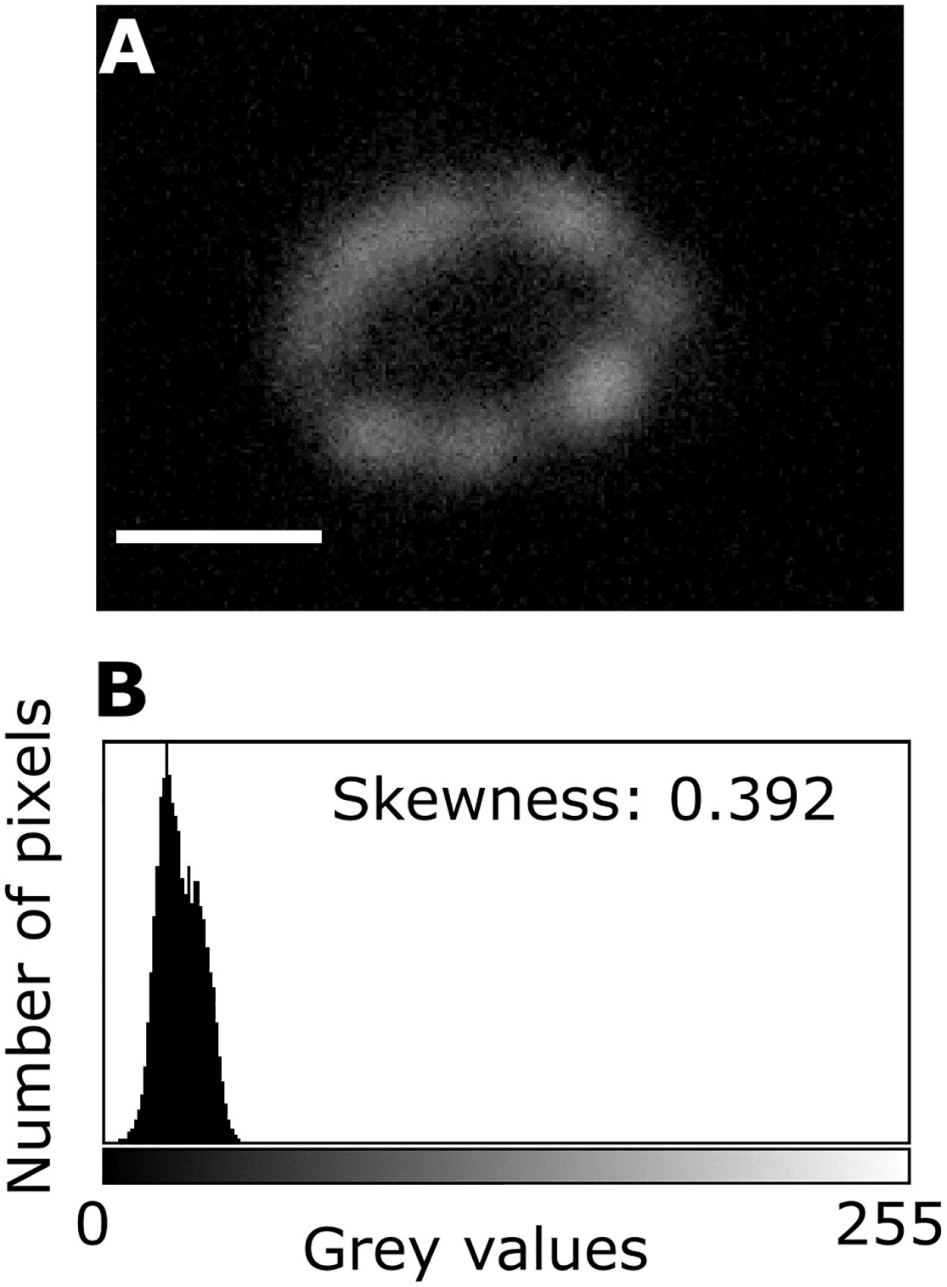
Skewness of a single *E. huxleyi* coccolith. A: *E. huxleyi* coccolith measured. B: Graph showing the distribution of pixels with various thickness in grey values for the coccolith in A. Because of the right skewed graph, the skewness value of the coccolith is positive, indicating that the thickness is asymmetrically skewed towards relatively thicker coccolith sections. White scale bar: 1. Note that the coccolith image has been altered after production of skewness graph to increase the visibility of the coccolith.

The CPR-method also allowed for the measurement of the coccolith aspect ratio and the skewness of the grey value distribution of a coccolith (Fig 2) in ImageJ. These measurements served as coccolith shape descriptors. The grey value skewness is a description of the distribution of thickness per pixel for a single coccolith, where a value of 0 signifies a symmetric distribution of pixel thickness, negative values signifies a left skewed distribution of pixel thickness (indicating a proportionally larger section of the coccolith is thinner compared to symmetrically distributed thickness), and positive values signifies a right skewed distribution of pixel thickness (indicating a proportionally larger section of the coccolith is thicker compared to symmetrically distributed thickness). Meanwhile, the aspect ratio describes the length to width ratio of the coccolith (i.e. roundness of a coccolith).

#### k_s_ calculation

Measured mass and length was used to calculate the k_s_ value for each coccolith. This was done by rearranging Eq 1 to

$$\begin{array}{c} k_{s}=\frac{m}{l^{3}\times d} \end{array}$$ (3)

where *m* is measured coccolith mass, *l* is measured coccolith length, and *d* is the density of calcite (= 2.71 g m^−3^).

### Statistical analysis

All statistics were done in R version 3.4.3 [50] using RStudio version 1.1.383. One-way ANOVA tests were performed on measured length, thickness, and mass to evaluate salinity related differences in all strains. In the case that the ANOVA test revealed a significant difference (*p* <0.05), a post-hoc Tukey HSD test was done to identify which of the samples differed. A linear regression analysis was done to evaluate the relationship between length and thickness in all samples. Linear regression models for each sample were evaluated using the qqPlot function in the R package “car” [51] and a Global Validation of Linear Model Assumptions (GVLMA) function in the R package “gvlma” [52]. GVLMA checks the model data for violations to the linear regression assumptions of linearity, homoscedascity, normality, and independence of individual measurements [53]. Data which violated one or more of the above assumptions were log-transformed before linear regression. In some regression models (three length versus mass models, five length versus thickness models, and four length versus skewness models) transformation did not fix the violations; in these cases the samples were left untransformed. Removal of one to six data points identified as violating the assumptions from the qqPlot function would fix the violations in these cases.

## Results

**Fig 3:**
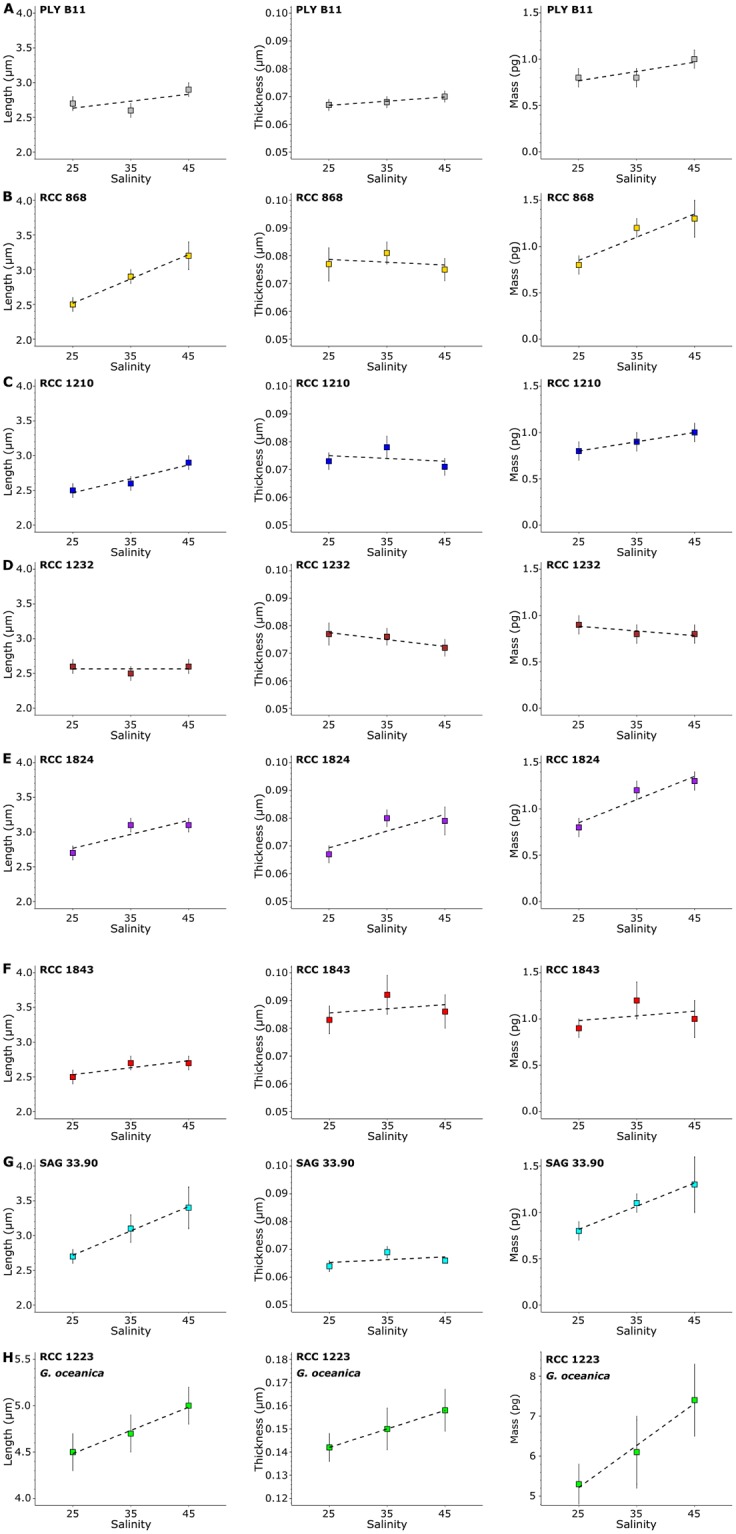
Mean values of measured coccolith length, mean thickness, and mass versus salinity. Each plot represents one strain: A: PLY B11 (*E. huxleyi*). B: RCC 868 (*E. huxleyi*). C: RCC 1210 (*E. huxleyi*). D: RCC 1232 (*E. huxleyi*). E: RCC 1824 (*E. huxleyi*). F: RCC 1843 (*E. huxleyi*). G: SAG 33.90 (*E. huxleyi*). H: RCC 1223 (*G. oceanica*). Vertical bars: 95% confidence interval of the mean value. Dashed line: Trend line of parameter with increasing salinity.

Two coccolithophore species, *E. huxleyi* and *G. oceanica*, were grown at salinities of 25, 34, and 44 to test the assumption that length and thickness increase at proportionate rates as suggested by [26]. Coccolith length, thickness, and mass of 30 coccoliths per sample were measured using the CPR-method. Mean *E. huxleyi* coccolith length ranged from 2.5 μm to 3.4 μm, while mean length of *G. oceanica* ranged from 4.5 μm to 5.1 μm (Table 3). Coccolith length increased statistically significantly from 25 to 44 salinity in *E. huxleyi* strains RCC 868, RCC 1210, RCC 1824, and SAG 33.90 (Table 4, Fig 3). In RCC 1210 and RCC 1824 the increase in length was 0.4 and in RCC 868 and SAG 33.90 the increase was 0.7 μm. A statistically significant length increase of 0.6 μm from 25 to 44 salinity was also seen in the *G. oceanica* strain RCC 1223 (Table 4, Fig 3H). In the *E. huxleyi* strains PLY B11, RCC 1232, and RCC 1843 the length increase was either not statistically significant or too small to be resolved. PLY B11 coccoliths grown at 25 and 34 salinity showed coccolith lengths of 2.53 μm and 2.57 μm, respectively, when measured in SEM images. This corresponds well with measured coccolith lengths in the LM samples from the same experiments.

**Table 3:** Mean coccolith length, thickness, mass, skewness of thickness distribution, and estimated k_s_ of all cultures. N: number of coccolith measured. Mean: measured mean value. CI: 95% confidence interval of measured mean.

Strain	Species	Salinity	N	Length (μm)		Mean thickness (μm)		Mass (pg)		Skewness		k_s_	
				Mean	CI	Mean	CI	Mean	CI	Mean	CI	Mean	CI
PLY B11	*E. huxleyi*	25	30	2.7	0.1	0.067	0.002	0.8	0.1	0.446	0.100	0.014	0.001
PLY B11		34	30	2.6	0.1	0.068	0.002	0.8	0.1	0.518	0.100	0.015	0.001
PLY B11		44	30	2.9	0.1	0.070	0.002	1.0	0.1	0.478	0.100	0.015	0.001
RCC 868	*E. huxleyi*	25	30	2.5	0.1	0.077	0.005	0.8	0.1	0.509	0.100	0.018	0.001
RCC 868		34	30	2.9	0.1	0.081	0.004	1.2	0.1	0.693	0.100	0.018	0.001
RCC 868		44	30	3.2	0.2	0.075	0.004	1.3	0.2	0.719	0.100	0.015	0.001
RCC 1210	*E. huxleyi*	25	30	2.5	0.1	0.073	0.003	0.8	0.1	0.503	0.100	0.018	0.001
RCC 1210		34	30	2.6	0.1	0.078	0.004	0.9	0.1	0.458	0.100	0.018	0.001
RCC 1210		44	30	2.9	0.1	0.071	0.003	1.0	0.1	0.604	0.100	0.015	0.001
RCC 1223	*G. oceanica*	25	30	4.5	0.2	0.142	0.006	5.3	0.5	0.868	0.100	0.021	0.001
RCC 1223		34	30	4.7	0.2	0.150	0.009	6.1	0.8	0.914	0.100	0.021	0.001
RCC 1223		44	30	5.1	0.2	0.158	0.009	7.4	0.8	0.802	0.100	0.021	0.001
RCC 1232	*E. huxleyi*	25	30	2.6	0.1	0.078	0.004	0.9	0.1	0.789	0.100	0.019	0.001
RCC 1232		34	30	2.5	0.1	0.076	0.003	0.8	0.1	0.559	0.100	0.019	0.001
RCC 1232		44	30	2.6	0.1	0.072	0.003	0.8	0.1	0.723	0.100	0.017	0.001
RCC 1824	*E. huxleyi*	25	30	2.7	0.1	0.067	0.003	0.8	0.1	0.820	0.100	0.014	0.001
RCC 1824		34	30	3.1	0.1	0.080	0.003	1.3	0.1	0.868	0.100	0.016	0.001
RCC 1824		44	30	3.1	0.1	0.079	0.005	1.3	0.1	0.771	0.100	0.016	0.001
RCC 1843	*E. huxleyi*	25	30	2.5	0.1	0.083	0.005	0.9	0.1	0.764	0.100	0.021	0.001
RCC 1843		34	30	2.7	0.1	0.092	0.006	1.2	0.2	0.696	0.100	0.022	0.001
RCC 1843		44	30	2.7	0.1	0.086	0.006	1.0	0.2	0.614	0.100	0.019	0.001
SAG 33.90	*E. huxleyi*	25	30	2.7	0.1	0.064	0.002	0.8	0.1	0.446	0.100	0.014	0.001
SAG 33.90		34	30	3.1	0.2	0.069	0.002	1.1	0.1	0.843	0.100	0.014	0.001
SAG 33.90		44	30	3.4	0.3	0.066	0.001	1.3	0.3	0.631	0.100	0.012	0.001

**Table 4:** One-way ANOVA and Tukey HSD results for evaluating the effect of salinity on mean coccolith length, thickness, and mass in each strain. SS: Sum of squares. df: degrees of freedom. MS: Mean square. F: F-value. Diff.: difference between measured means. *p*: probability of falsely rejecting the null hypothesis. CL: 95% confidence level for the difference of means in a Tukey HSD test. Adjusted *p*: *p* after adjustment for multiple comparisons. “*” indicates significant *p* values <0.05. Differences that are significant according to ANOVA and Tukey HSD and also greater or equal to twice the measurement standard uncertainty (±0.2 μm for length, ±0.007 μm for thickness, and ±14-18% for mass) are shown in bold.

Strain	Parameter	ANOVA						Tukey HSD				
			SS	df	MS	F	*p*	Salinity	Diff.	Lower CL	Upper CL	Adjusted *p*
PLY B11	Length (μm)	Salinity	1.53	2	0.76	8.33	<0.01*	34-25	-0.1	-0.2	0.1	0.82
		Residuals	7.98	87	0.09			44-25	0.2	0.1	0.4	<0.01*
								44-34	0.3	0.1	0.5	<0.01*
	Thickness (μm)	Salinity	0.00	2	0.00	1.37	0.26	-	-	-	-	-
		Residuals	0.00	87	0.00							
	Mass (pg)	Salinity	1.39	2	0.69	12.40	<0.01*	34-25	0.0	-0.1	0.1	1.00
		Residuals	4.87	87	0.06			**44-25**	**0.2**	**0.1**	**0.4**	<0.01*
								**44-34**	**0.2**	**0.1**	**0.4**	<0.01*
RCC 868	Length (μm)	Salinity	7.15	2	3.57	21.64	<0.01*	**34-25**	**0.4**	**0.2**	**0.7**	<0.01*
		Residuals	14.37	87	0.17			**44-25**	**0.7**	**0.4**	**0.9**	<0.01*
								44-34	0.3	0.0	0.5	0.04*
	Thickness (μm)	Salinity	0.00	2	0.00	1.99	0.14	-	-	-	-	-
		Residuals	0.01	87	0.00							
	Mass (pg)	Salinity	4.54	2	2.27	13.73	<0.01*	**34-25**	**0.4**	**0.1**	**0.6**	<0.01*
		Residuals	14.39	87	0.17			**44-25**	**0.5**	**0.3**	**0.8**	<0.01*
								44-34	0.1	-0.1	0.4	0.40
RCC 1210	Length (μm)	Salinity	1.95	2	0.98	7.45	<0.01*	34-25	0.1	-0.1	0.3	0.63
		Residuals	11.40	87	0.13			44-25	0.4	0.1	0.6	<0.01*
								**44-34**	**0.3**	**0.0**	**0.5**	0.02*
	Thickness (μm)	Salinity	0.00	2	0.00	4.96	<0.01*	34-25	0.005	-0.001	0.010	0.14
		Residuals	0.007	87	0.000			44-25	-0.002	-0.008	0.003	0.47
								44-34	-0.007	-0.012	-0.002	<0.01*
	Mass (pg)	Salinity	0.73	2	0.36	3.76	0.03*	34-25	0.1	-0.1	0.3	0.34
		Residuals	8.39	87	0.10			44-25	0.2	0.0	0.4	0.02*
								*44-34*	0.1	-0.1	0.3	0.38
RCC 1223	Length (μm)	Salinity	4.41	2	2.21	8.02	<0.01*	34-25	0.2	-0.1	0.6	0.22
		Residuals	23.93	87	0.28			**44-25**	**0.6**	**0.2**	**0.9**	<0.01*
								44-34	0.4	0.0	0.6	0.06
	Thickness (μm)	Salinity	0.00	2	0.00	4.18	0.02*	34-25	0.008	-0.006	0.021	0.36
		Residuals	0.043	87	0.000			**44-25**	**0.016**	**0.003**	**0.030**	0.01*
								44-34	0.008	-0.005	0.022	0.29
	Mass (pg)	Salinity	72.60	2	36.32	9.20	<0.01*	34-25	0.8	-0.3	2.1	0.21
		Residuals	343.60	87	3.95			**44-25**	**2.1**	**1.0**	**3.4**	<0.01*
								44-34	1.3	0.1	2.5	0.03
RCC 1232	Length (μm)	Salinity	0.03	2	0.02	0.15	0.87	-	-	-	-	-
		Residuals	8.89	87	0.10							
	Thickness (μm)	Salinity	0.00	2	0.00	2.17	0.12	-	-	-	-	-
		Residuals	0.01	87	0.00							
	Mass (pg)	Salinity	0.09	2	0.05	0.54	0.59	-	-	-	-	-
		Residuals	7.24	87	0.08							
RCC 1824	Length (μm)	Salinity	2.16	2	1.08	14.43	<0.01*	**34-25**	**0.4**	**0.2**	**0.5**	<0.01*
		Residuals	6.50	87	0.08			**44-25**	**0.4**	**0.2**	**0.5**	<0.01*
								44-34	0.0	-0.2	0.2	1.00
	Thickness (μm)	Salinity	0.00	2	0.00	14.28	<0.01*	34-25	0.013	0.006	0.018	<0.01*
		Residuals	0.01	87	0.00			44-25	0.012	0.006	0.018	<0.01*
								44-34	-0.001	-0.007	0.006	0.98
	Mass (pg)	Salinity	3.88	2	1.94	24.69	<0.01*	**34-25**	**0.5**	**0.3**	**0.6**	<0.01*
		Residuals	6.84	87	0.08			**44-25**	**0.5**	**0.3**	**0.6**	<0.01*
								44-34	0.0	-0.1	0.2	0.93
RCC 1843	Length (μm)	Salinity	1.08	2	0.54	3.723	0.03*	34-25	0.2	0.0	0.5	0.04*
		Residuals	12.64	87	0.15			44-25	0.2	0.0	0.5	0.07
								44-34	0.0	-0.3	0.2	0.97
	Thickness (μm)	Salinity	0.00	2	0.00	2.31	0.11	-	-	-	-	-
		Residuals	0.02	87	0.00							
	Mass (pg)	Salinity	1.35	2	0.68	3.38	0.04*	**34-25**	**0.3**	**0.0**	**0.6**	0.03*
		Residuals	17.40	87	0.20			44-25	0.1	-0.1	0.4	0.38
								44-34	-0.2	-0.4	0.1	0.4
SAG 33.90	Length (μm)	Salinity	6.77	2	3.38	12.00	<0.01*	**34-25**	**0.4**	**0.1**	**0.7**	0.02*
		Residuals	24.53	87	0.28			**44-25**	**0.7**	**0.3**	**1.0**	<0.01*
								44-34	0.3	0.0	0.6	0.09
	Thickness (μm)	Salinity	0.00	2	0.00	8.92	<0.01*	34-25	0.005	0.002	0.008	<0.01*
		Residuals	0.00	87	0.00			44-25	0.002	-0.001	0.005	0.22
								44-34	-0.003	-0.006	0.000	0.04*
	Mass (pg)	Salinity	4.46	2	2.23	10.17	<0.01*	**34-25**	**0.3**	**0.0**	**0.6**	0.03*
		Residuals	19.06	87	0.22			**44-25**	**0.5**	**0.3**	**0.8**	<0.01*
								44-34	0.2	-0.1	0.5	0.136

Mean coccolith thickness ranged from 0.064 to 0.092 in *E. huxleyi* and 0.142 μm to 0.158 μm in *G. oceanica*. A statistically significant 0.016 μm increase in thickness from 25 to 44 salinity is seen in the *G. oceanica* strain. Measured mean coccolith mass ranged from 0.8pg to 1.3pg in *E. huxleyi*, and from 5.3pg to 7.4pg in *G. oceanica*. Mass increased statistically significantly with salinity in the *E. huxleyi* strains PLY B11, RCC 868, RCC 1824, and SAG 33.90, and in the *G. oceanica* strain. In the *E. huxleyi* strains RCC 1210, RCC 1232, and RCC 1843 the mass increase was either not statistically significant, too small to be resolved, or in the case of RCC 1843 only seen between 25 and 34 salinity.

**Fig 4:**
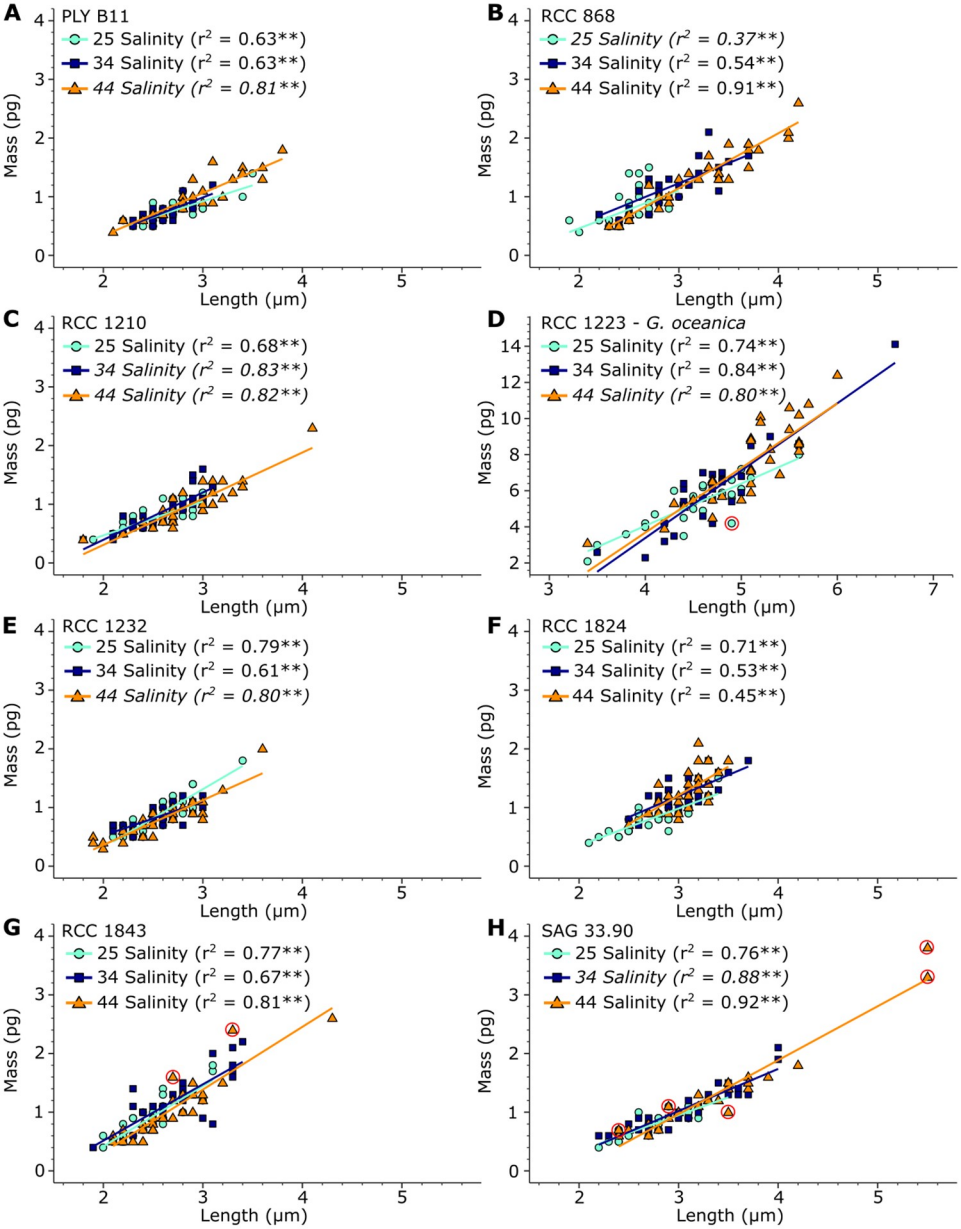
Scatter plots of coccolith length versus coccolith mass for all strains and salinities analysed. Cyan dots: coccoliths cultured under 25 salinity conditions. Blue squares: coccoliths cultured under 34 salinity conditions. Orange triangles: coccoliths cultured under 44 salinity conditions. Colored lines: linear regression lines for each sample. “*” indicates a significant relationship with *p*<0.05, while “**” indicates a significant relationship with *p*<0.01. r^2^ values in italics were obtained after log-transformation. Red circles show data points that were found to be influential according to the R functions gvlma and qqPlot.

**Fig 5:**
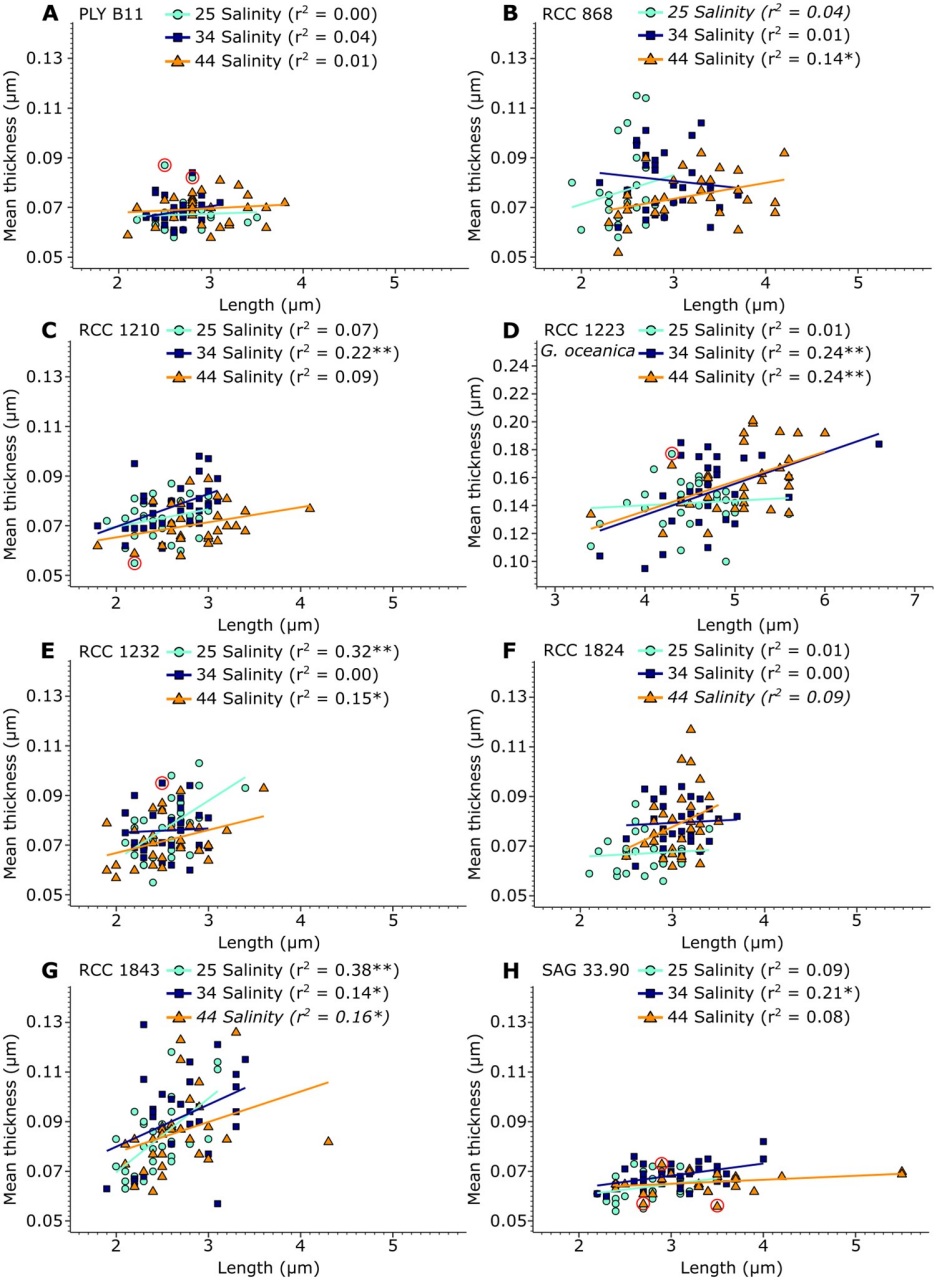
Scatter plots of coccolith length versus coccolith thickness for all strains and salinities analysed. Cyan dots: coccoliths cultured under 25 salinity conditions. Blue squares: coccoliths cultured under 34 salinity conditions. Orange triangles: coccoliths cultured under 44 salinity conditions. Colored lines: linear regression lines for each sample. “*” indicates a significant relationship with *p*<0.05, while “**” indicates a significant relationship with *p*<0.01. r^2^ values in italics were obtained after log-transformation. Red circles show data points that were found to be influential according to the R functions gvlma and qqPlot.

Coccolith length and mass was positively related in all 24 coccolith samples, with r^2^ values ranging from 0.37 to 0.92 and *p* <0.05 (Fig 4). In contrast, a statistically significant positive linear relationship between coccolith length and thickness was only found in 8 of 21 *E. huxleyi* experiments, with r^2^ values ranging from 0.14 to 0.38, and in two of three *G. oceanica* experiments (RCC 1223), with a r^2^ of 0.24 (Fig 5). In the remaining 14 samples the relationship is statistically not significant, with r^2^ values ranging from 0.00 to 0.13. In one sample, SAG 33.90 grown at 44 salinity (Fig 5H), the relationship between length and thickness became significant after violations to the linear regression model were removed, though the relationship remained weak with an r^2^ of 0.15.

## Discussion

In this study, the effects of salinity on coccolith thickness and mass in the two species of *E. huxleyi* and *G. oceanica* were investigated for the first time. This study revealed important insights into the relationship between coccolith length and thickness in these species, and the implications of these insights on the k_s_ model, as discussed in detail below.

### Salinity effects on *E. huxleyi* coccolith length

The salinity effect on *E. huxleyi* coccolith length is well documented from both culture, plankton, and sediment studies [34–39]. In this study, salinity also affected coccolith length in the *E. huxleyi* strains RCC 868, RCC 1210, RCC 1824, and SAG 33.90. However, the other strains, PLY B11, RCC 1232, and RCC 1843, did not increase significantly in length with thickness. This is consistent with [35], who only found an increase in length with salinity in two out of three strains. Furthermore, [36] found that coccoliths sampled from areas with salinities outside a typical open ocean range (33-38 salinity) deviated from the typical salinity response. For this reason, [37] hypothesized that open ocean populations of *E. huxleyi* display a more marked morphological response to salinity than coastal populations, as the larger salinity fluctuations in coastal regions may have led to different adaptations to changing salinity in coastal *E. huxleyi* populations. In this regard it is interesting to note that while most strains used in this study were isolated from a coastal region (Fig 1), the strain isolated furthest from the coast, RCC 868, was among the two *E. huxleyi* strains with the greatest increase in length with salinity.

### Relationship between *E. huxleyi* coccolith length, thickness and shape

Salinity did not affect *E. huxleyi* coccolith thickness in this study, even when coccolith length increased. Coccolith length and thickness therefore do not seem to be closely coupled parameters in *E. huxleyi*, in contrast to prior assumptions (e.g. [26, 54, 55]). An inspection of the relationship between length and thickness within each individual experiment confirms this. Coccolith length and thickness are not, or only weakly, related (when significant, r^2^ values <0.4) in the *E. huxleyi* experiments (Fig 5). Coccolith size increases thus appears to be allometric, meaning that coccolith length and thickness increase at disproportionate rates. This stands in contrast to the k_s_ model, which assumes isometric increases in coccolith size, i.e. that coccolith length and thickness increase at proportionate rates [26].

**Fig 6:**
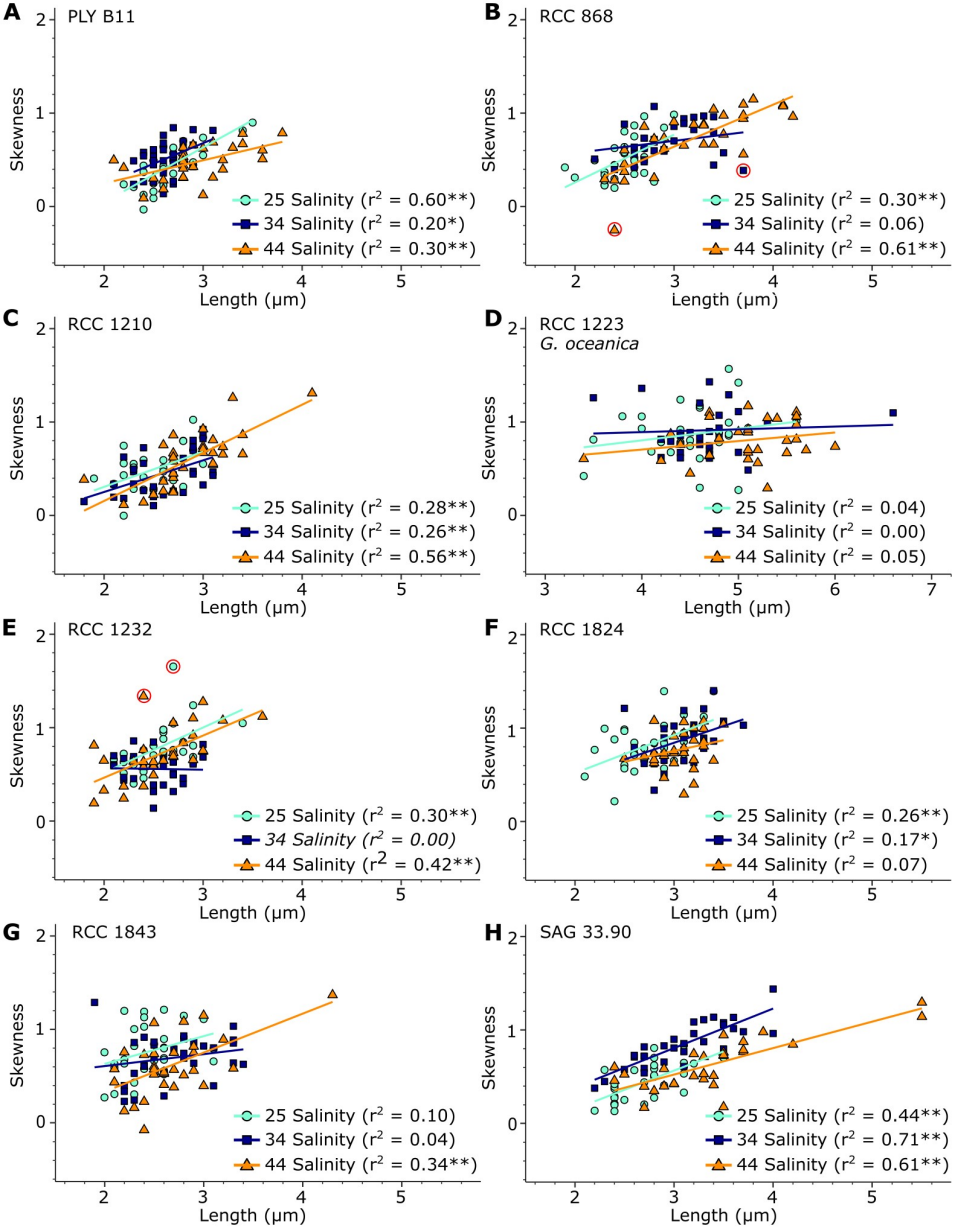
Coccolith length versus distribution of thickness in terms of skewness for individual coccoliths in each strain and salinity culture. Cyan dots: coccoliths cultured under 25 salinity conditions. Blue squares: coccoliths cultured under 34 salinity conditions. Orange triangles: coccoliths cultured under 44 salinity conditions. Colored lines: linear regression lines for each sample. “*” indicates a significant relationship with *p*<0.05, while “**” indicates a significant relationship with *p*<0.01. r^2^ values in italics were obtained after log-transformation. Red circles show data points that were found to be influential according to the R functions gvlma and qqPlot.

If *E. huxleyi* coccoliths increase in size allometrically, coccolith shape by definition changes as the coccolith grows. This is seen in several experiments where coccolith length and skewness are positively related (Fig 6). This indicates that the thickness of different sections/parts of the coccolith vary in relation to each other, and confirms that the coccolith shape changes as the coccolith size increases. Neither thickness or skewness increased with coccolith length in the 34 salinity experiments of RCC 868 and RCC 1232 or the 44 salinity experiment of the RCC 1824 strain, however. Instead, the strain RCC 1232 showed a significant positive relationship between length and aspect ratio (indicative of roundness) when cultured under 34 salinity, indicating width increased at a smaller rate than length in this culture, thus changing the coccolith shape. A significant relationship between length and aspect ratio was also seen in the strains RCC1824 and SAG 33.90 when cultured under 34 salinity.

### Salinity effect on *E. huxleyi* coccolith mass

**Fig 7:**
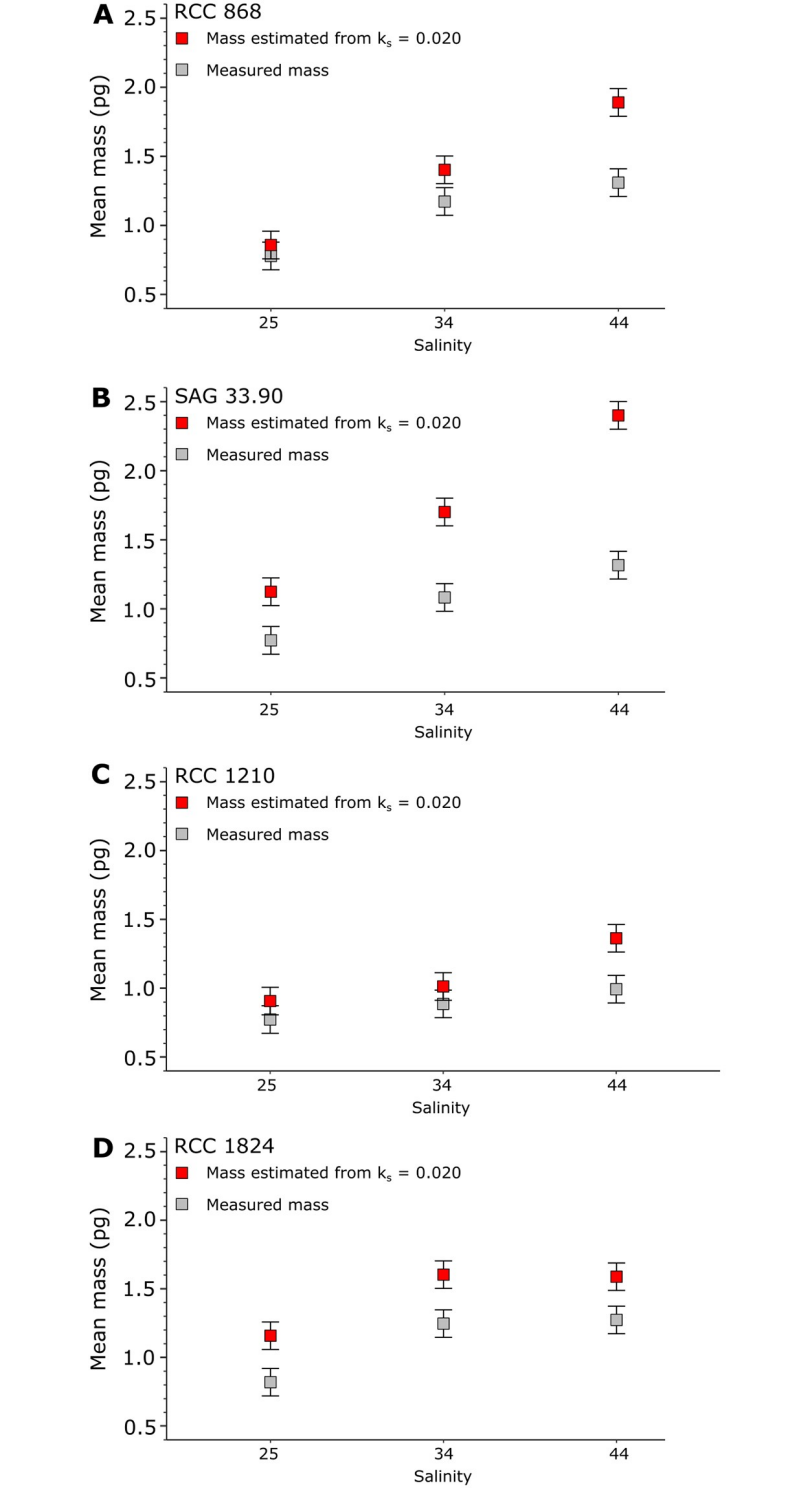
Coccolith mass versus salinity in *E. huxleyi* strains RCC 868 (A), SAG 33.90 (B), RCC 1210 (C), and RCC 1824 (D). Grey squares: measured mean mass. Red squares: Estimated mean mass using the recommended k_s_ value of 0.020 for *E. huxleyi* [26]. Vertical bars: 0.1pg standard error of each measurement.

Though coccolith length increased with salinity in several *E. huxleyi* strains (Fig 3), coccolith mass did not increase in the manner expected from the k_s_ model. The k_s_ value of 0.02 recommended for *E. huxleyi* Type A coccoliths [26] was too high, and the increase in mass with increasing length was smaller than expected from the k_s_ model, leading to a significant bias as the difference in estimated mass from the k_s_ model increased with increasing coccolith length (Fig 7). For example, over the 25 to 44 salinity range, measured coccolith mass increased by 0.5pg in both RCC 868 and SAG 33.90, but the expected mass increase from a constant k_s_ factor of 0.02 is ~1.0pg and ~1.3pg, respectively (Fig 7A and 7B). While at 25 salinity the k_s_ model overestimated coccolith mass in the two strains by 12% and 38% compared to measured mass, the k_s_ model overestimated RCC 868 and SAG 33.90 coccolith mass by 46% and 85% at 44 salinity. In the *E. huxleyi* strain RCC 1824 the rate of increase in coccolith mass with increasing length was more in line with the k_s_ model, though a constant k_s_ factor of 0.02 still overestimates mass (Fig 7D). Furthermore, the salinity response in RCC 1824 is not consistent over the salinity range of 25 to 44 salinity. Neither coccolith length, mass, or thickness increased from 34 to 44 salinity.

The smaller mass increase with salinity in *E. huxleyi* than expected from the k_s_ model is because thickness did not increase with salinity in these strains, which highlights the importance of considering both coccolith length and thickness in coccolith mass estimations. This does not mean that salinity does not affect coccolith mass, however, as suggested by [17]. In most *E. huxleyi* strains, a significant increase in coccolith length with salinity was also accompanied by a significant increase in coccolith mass, though as mentioned the increase was smaller than expected from the k_s_ model (Table 4). [34] similarly reported an increase in coccolith mass with salinity.

#### *E. huxleyi* morphotypes

The different responses to increasing salinity in *E. huxleyi* and *G. oceanica* in this study suggest that the coccolith thickness response to increasing salinity is genetically determined. Several different morphotypes are described for *E. huxleyi*, potentially representing separate genotypes [56–59]. This study only analysed *E. huxleyi* Type A, and [35] and [38] also focused on Type A in their investigation of coccolith length under different salinities. [39], however, reported changing coccolith length with changing salinity in a strain of *E. huxleyi* they identified as Type B/C, suggesting that the coccolith length response to changing salinity is consistent between morphotypes. The consistent salinity response in plankton and sediment samples [36, 37] further support this suggestion. It can not be excluded that other morphotypes have a different thickness response than reported for Type A in this study. However, Type A does not appear to represent one phylogenetic group [59], so the consistent lack of a relationship between thickness and salinity in all *E. huxleyi* strains may represent a species-wide response.

### Effects of other environmental variables on *E. huxleyi* coccolith morphology and mass

This study found that salinity affects *E. huxleyi* coccolith morphology in terms of length and mass, but not thickness. Salinity is not the only environmental parameter with reported effects on *E. huxleyi* coccolith morphology, however. Studies have reported effects of temperature [39, 60, 61], nutrient limitation [62, 63], and CO_2_ [64, 65] on coccolith morphology in *E. huxleyi*. The temperature effect, though, varies greatly between studies, and different studies have for example reported either an increase [61], decrease [39, 60], or no change [38] in coccolith length with increasing temperature. These inconsistent results may indicate that the morphological response in *E. huxleyi* to changing temperature is strain-specific, as it is for salinity (this study and [35]). Alternatively, different culture conditions between studies may have led to inconsistent results, as different culture conditions is known to affect calcite production in *E. huxleyi* under changing CO_2_ conditions (e.g. [66, 67]). Culture conditions may possibly affect the temperature response as well.

[63] reported a decrease in coccolith mass with N limitation and increase in coccolith mass with P limitation, using atomic absorption spectrometry to measure Ca content per coccolith. This method does not allow for separate evaluation of coccolith length, thickness, and mass of individual coccoliths like the CPR-method used in this study. However, under P limitation coccolith length did not change [63], indicating that the increase in coccolith mass was due to an increase in thickness rather than length. Under N limitation, on the other hand, both coccolith habitus and length changed, so that the relative impact of coccolith length and thickness on coccolith mass under N limitation can not be determined. If various environmental variables influence coccolith length and thickness differently, then coccolith length and thickness may both be valuable parameters to disentangle multiple effects on coccolith mass in plankton and sediment samples.

#### Growth rate

Previous studies have reported an effect of salinity on growth rates in *E. huxleyi* [34, 39]. In these studies, growth rates have either decreased with decreasing salinity [39] or shown an optimum curve with different optimal values between strains [34]. In this study no consistent relationship between salinity and growth rate could be seen among strains. Only four strains showed an optimum curve as reported in [34], and there was no consistent decrease in growth rate with salinity in any strain.

**Fig 8:**
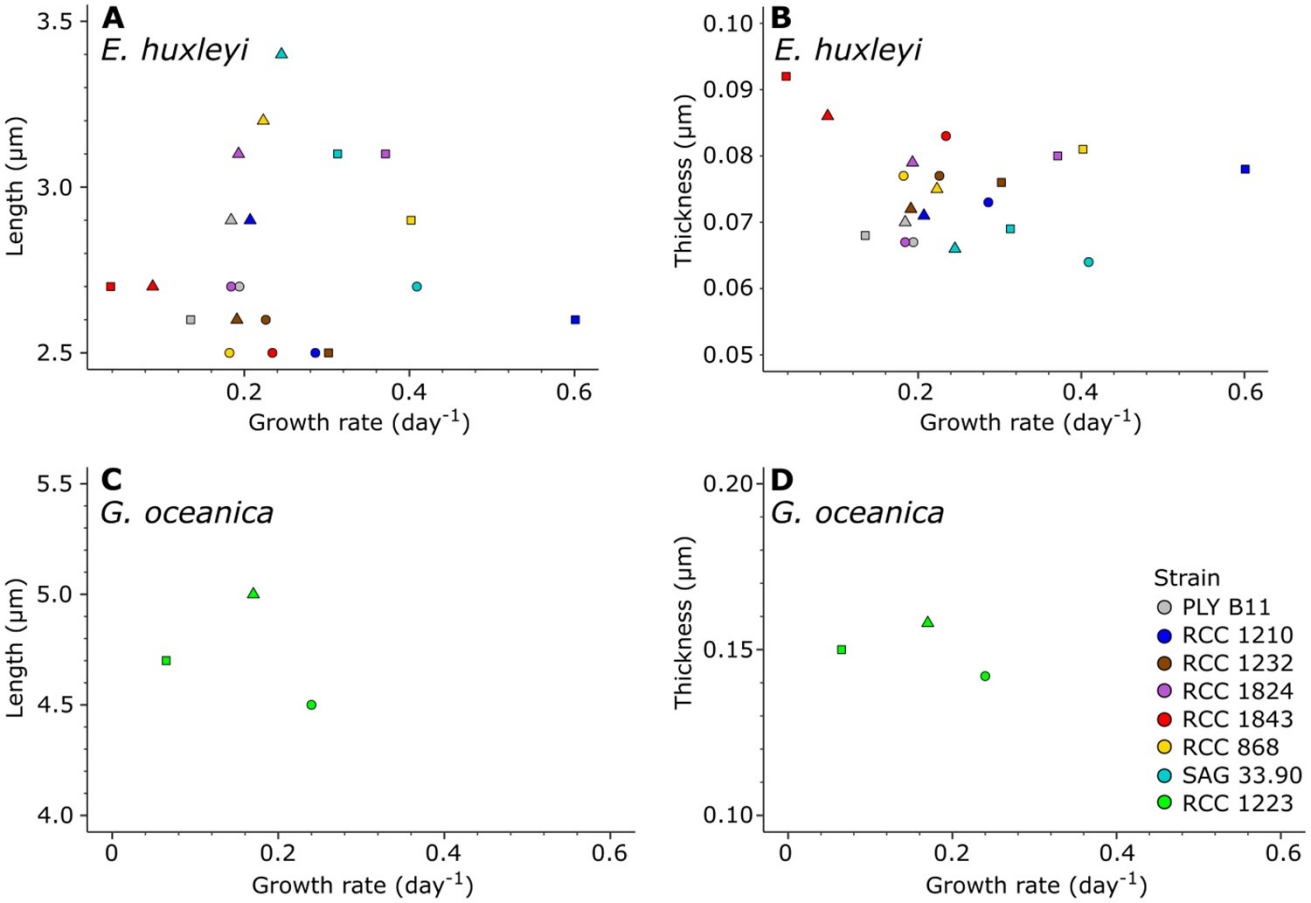
Growth rate versus coccolith length and thickness in *E. huxleyi* and *G. oceanica* strains in this study. A: Growth rate versus coccolith length for all *E. huxleyi* strains. B: Growth rate versus coccolith thickness for all *E. huxleyi* strains. C: Growth rate versus coccolith length for the *G. oceanica* strain. D: Growth rate versus coccolith thickness for the *G. oceanica* strain. Circles: cultures grown at 25 salinity. Squares: cultures grown at 34 salinity. Triangles: cultures grown at 44 salinity.

Growth rate has been suggested to be linked to cell geometry in coccolithophores in terms of cell size and number of coccoliths per cell [68]. A similar link between coccolith morphology and growth rate would be of great interest in determining past growth rates from the geological record [61]. There was, however, no clear link between growth rate and coccolith length or thickness in the present study (Fig 8).

### Salinity effects on *G. oceanica* coccolith morphology

In contrast to *E. huxleyi*, *G. oceanica* coccolith length and thickness both increased with salinity. This is the first report that the size of *G. oceanica* coccoliths are affected by salinity, as they also are by temperature [69]. However, [69] reported an increase in coccolith length of the morphotype *Gephyrocapsa* Equatorial, whereas the present study showed an increase in coccolith length of the *Gephyrocapsa* Larger morphotype. Furthermore, there was a coccolith mass increase with salinity which was consistent with a constant k_s_ factor of ~0.02. *G. oceanica* coccolith size thus increased in an isometric fashion with increasing salinity. However, the ~0.02 k_s_ values for *G. oceanica* in this study is significantly smaller than the k_s_ value of 0.05 reported by [26]. *G. oceanica* coccolith mass in this study was therefore less than expected from these k_s_ values.

**Fig 9:**
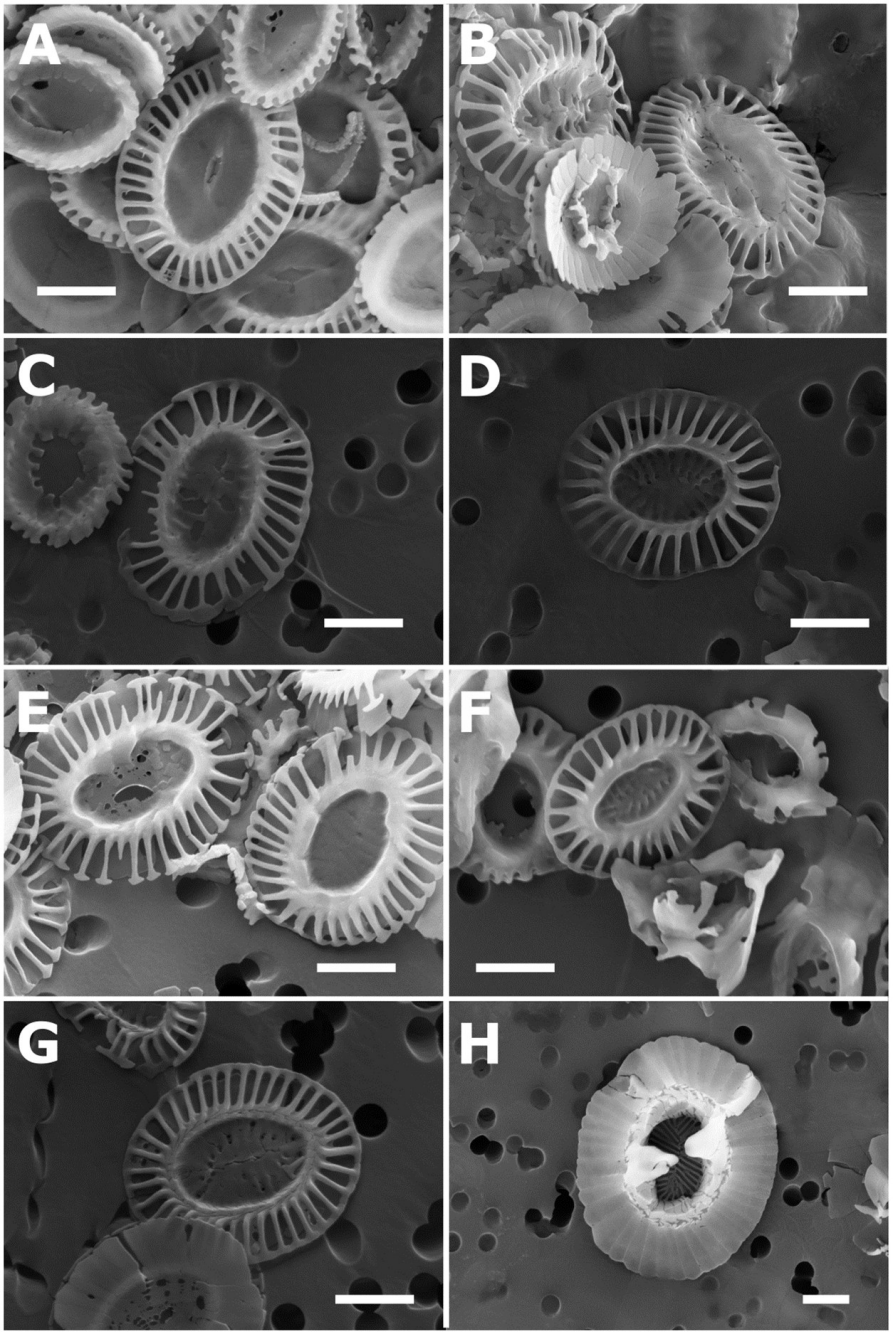
Images of typical coccoliths from each strain. *E. huxleyi*: A-G: A: PLY B11. B: RCC 868. C: RCC 1210. D: RCC 1232. E: RCC 1824. F: RCC 1843. G: SAG 33.90. *G. oceanica*: H: RCC 1223. White bar: Scale bar (1 μm).

The low k_s_ value of *G. oceanica* in this study may be explained by malformation. Malformation is a common phenomenon in coccolithophore cultures [70–72], and many *G. oceanica* coccoliths in this study were malformed (Fig 9H). However, malformation does not appear to have affected the relationship between salinity and length or thickness in *G. oceanica*.

## Conclusion

This study confirmed that salinity affects coccolith length but not coccolith thickness in *E. huxleyi*. As a result, coccolith mass did not increase with salinity at the same rate as it would be expected from an isometric coccolith growth model proposed by [26]. Therefore, the k_s_ factor reported by [26] should be used with caution for the calculation of *E. huxleyi* coccolith mass. On the other hand, the length and thickness of *G. oceanica* increased with increasing salinity. Coccolith mass calculations for *G. oceanica* based on a k_s_ value of 0.02 compared well with measured mass at all salinities, indicating a constant rate of increase in both length and thickness with salinity in this species. However, [26] reported a k_s_ value of 0.05 for this species, which would lead to a ~140% overestimation of mass if applied to *G. oceanica* here. This study revealed an important complication in approaches attempting to estimate coccolith mass from length. Different relationships between coccolith length and thickness means that coccolith mass can not be accurately estimated from coccolith length alone.

## Supporting information

S1 Appendix A csv file containing all data is supplied with this article. (CSV)
